# EphA receptors regulate prostate cancer cell dissemination through Vav2–RhoA mediated cell–cell repulsion

**DOI:** 10.1242/bio.20146601

**Published:** 2014-05-02

**Authors:** Jennifer Batson, Lucy Maccarthy-Morrogh, Amy Archer, Helen Tanton, Catherine D. Nobes

**Affiliations:** 1 School of Physiology and Pharmacology, University of Bristol, Bristol BS8 1TD, UK; 2 School of Biochemistry, University of Bristol, Bristol BS8 1TD, UK

**Keywords:** Eph receptors, Contact inhibition of locomotion, Cell migration, Prostate cancer, Rho GTPases, Vav2

## Abstract

Metastatic prostate cancer cells display EphB receptor-mediated attraction when they contact stromal fibroblasts but EphA-driven repulsion when they contact one another. The impact of these ‘social’ interactions between cells during cancer cell invasion and the signalling mechanisms downstream of Eph receptors are unclear. Here we show that EphA receptors regulate prostate cancer cell dissemination in a 2D dispersal assay and in a 3D cancer cell spheroid assay. We show that EphA receptors signal via the exchange factor Vav2 to activate RhoA and that both Vav2 and RhoA are required for prostate cancer cell–cell repulsion. Furthermore, we find that in EphA2/EphA4, Vav2 or RhoA siRNA-treated cells, contact repulsion can be restored by partial microtubule destabilisation. We propose that EphA–Vav2–RhoA-mediated repulsion between contacting cancer cells at the tumour edge could enhance their local invasion away from the primary tumour.

## INTRODUCTION

To metastasise, carcinoma cells in primary tumours must first break down their epithelial cell–cell junctions and break away from the tumour mass. Cancer cells then invade locally through surrounding extracellular matrix and stromal tissues towards blood or lymphatic vessels (Friedl and Alexander, 2011). During invasion through the tumour microenvironment and surrounding stroma, migrating cancer cells come into contact with both cancer cells and non-cancer cells. The ‘social’ interactions between these contacting cells have been suggested to influence their invasive behaviour (Abercrombie, 1979; Veselý and Weiss, 1973). Many migrating malignant cells, including prostate cancer cells, exhibit cell–cell repulsion, also known as contact inhibition of locomotion (CIL), when they contact one another (Abercrombie, 1970; Abercrombie and Heaysman, 1954; Astin et al., 2010; Batson et al., 2013). This has been well described on 2D surfaces *in vitro* where, upon contact, migrating cells stop moving, retract their protrusions, repolarise and reinitiate migration in a new direction to move away from one another into free space. By contrast, many metastatic malignant cells display the opposite behaviour – attractive migration – when they contact stromal cells such as fibroblasts or endothelial cells (Astin et al., 2010). This behaviour often results in the migrating cancer cell crawling beneath its stromal neighbour (Astin et al., 2010).

Contact-induced repulsive and attractive migration have been known about for almost 60 years and have recently been shown to occur *in vivo* (Carmona-Fontaine et al., 2008; Davis et al., 2012; Stramer et al., 2010; Moore et al., 2013). However, the molecular mechanisms involved and their roles in cancer cell dissemination, invasion and metastasis are not well understood. Recent work has shown that cancer cell migration following cell–cell contact can be regulated by a balance between repulsive EphA and attractive EphB receptor signalling (Astin et al., 2010) and thus is dependent on the relative level of ephrin-A and ephrin-B ligand and EphA and EphB receptor expression on the two confronting cells.

Ephrin type-A receptors and ephrin type-B receptors (Eph receptors) and their ephrin ligands have well described roles in vascular development, tissue boundary formation and axon guidance (Kullander and Klein, 2002; Pasquale, 2008). Both Eph receptor and ephrin ligand are membrane-bound and interact upon direct cell–cell contact leading to bidirectional signalling events in both cells. Eph–ephrin interactions are known to regulate cell morphology, adhesion and migration by signalling to the actin cytoskeleton, particularly via their effects on Rho GTPases (Noren and Pasquale, 2004). In many cell types microtubule polymerisation dynamics and polarisation are also important for cell motility, and microtubule dynamics have been shown to be required for the front–rear switch in polarity required for cell contact driven cell–cell repulsion (Kadir et al., 2011; Moore et al., 2013).

Eph receptor expression is frequently misregulated during tumour progression and EphA2 overexpression is associated with poor prognosis in prostate cancer patients (Lin et al., 2012; Zeng et al., 2003). EphB-mediated attractive migration of advanced cancer cells, as they contact stromal cells, has been suggested to increase their invasive capacity through the surrounding stroma (Astin et al., 2010). Here we have investigated whether, in addition, repulsive EphA receptor signalling can regulate local invasion away from the primary tumour mass. Using 2D and 3D models of cancer cell dispersal we have analysed the role of EphA receptors in cancer cell dissemination. In doing so, we further uncover the signalling mechanisms driving EphA-mediated cell–cell repulsion and find that signalling from EphA receptors, via the guanine nucleotide exchange factor (GEF) Vav2 to activate RhoA, can stimulate cancer cell–cell repulsion.

## RESULTS

### EphA2/EphA4 regulate prostate cancer cell dissemination and invasion

**Fig. 1.**
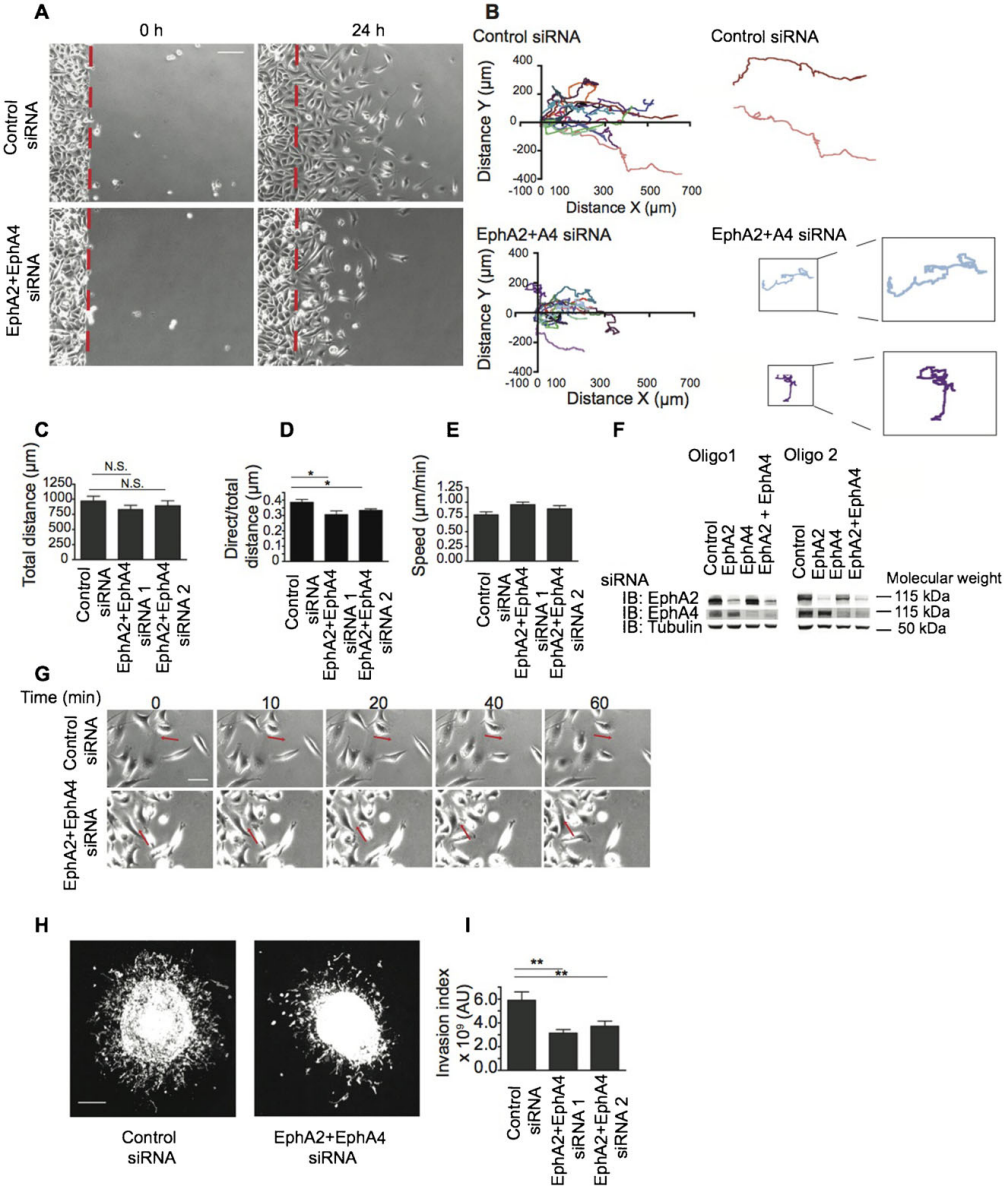
EphA2/EphA4 regulate prostate cancer cell dissemination and invasion. (A) Representative images from time-lapse movies at 0 h and 24 h after removal of silicon inserts. Cells were serum starved and treated with HGF 24 h prior to insert removal. Red dotted line indicates the starting position of cells at 0 h. (B) Cells were tracked over 24 h from the edge of the cancer cell population. Each colour is a track of an individual cell. Enlargement of cell tracks to show meandering migration of EphA2 + EphA4 siRNA-treated cells. (C) Quantification of the total distance and (D) the direct distance between the first and last point relative to the total distance migrated. (E) Quantification of cell speed. (F) Immunoblotting to show knockdown efficiency. (G) Stills from timelapse movies show examples of cell–cell repulsion between control cells and lack of repulsion in EphA2 + EphA4 siRNA-treated cells (supplementary material Movies 1 and 2). 0 min is the time at which cells came into contact. Arrows indicate direction of migration. (H) Confocal images of phalloidin staining of control siRNA or EphA2 + EphA4 siRNA-treated prostate cancer spheroids 6 days after injection into collagen gels. (I) Invasion index was quantified by thresholding confocal images in imageJ to make a binary image and multiplying the average distance from the tumour spheroid by the number of invaded cells (Nyström et al., 2005). Data are from 4 independent experiments. One asterisk indicates P<0.05, two asterisks indicate P<0.01, N.S.; not significant difference as determined by an unpaired Student's t-test. AU represents arbitrary units. Scale bars: 100 µm (A), 50 µm (G), 200 µm (H).

Our previous studies have shown that CIL and cell–cell repulsion in prostate cancer cells depend on EphA2 and EphA4 since knockdown of these receptors led to a loss of repulsion and failure of CIL (Astin et al., 2010; Batson et al., 2013; see also Fig. 6B). CIL does not only involve inhibition of forward migration but importantly also redirects migration away from the cell–cell collision site towards free space. CIL has recently been shown to define embryonic patterning of haemocytes in developing *Drosophila* embryos such that cells distribute uniformly throughout the embryo through repulsive interactions (Davis et al., 2012). In addition, Par3 – a mediator of CIL in neural crest cells – is required for neural crest cell dispersal in *Xenopus* embryos (Moore et al., 2013). We hypothesise that, in addition to driving embryonic cell dispersal during development, contact repulsion during CIL might also drive cancer cell dispersal from a tumour mass. To investigate the possible role of EphA/ephrin-A signalling in cancer cell dissemination, we seeded PC-3 cells into silicon inserts and removed the insert to create a cell population surrounded by free space. We then imaged and tracked cancer cell migration over 24 h. Control cells migrated significantly further from the cell population than did EphA2/EphA4 siRNA-treated cells (Fig. 1A). Tracking of individual cells (Fig. 1B) and quantification of total distance travelled (Fig. 1C) and the direct distance between the starting and finishing point of cell migration (Fig. 1D) showed that EphA2/EphA4 knockdown cells covered as much distance as control cells but they meandered back on themselves and in some cases re-entered the cancer cell population rather than continuing to migrate outwards into free space (Fig. 1G; supplementary material Movies 1 and 2). Migratory speed was not reduced in EphA2/EphA4 siRNA-treated cells compared to control siRNA-treated cells (Fig. 1E). Knockdown levels for control and EphA2/EphA4 siRNA-treated cells are shown 4 days after transfection but protein levels were reduced for up to 7 days (Fig. 1F and data not shown).

To investigate whether this difference in migration in 2D correlates with differences in invasion in 3D we used a cancer cell spheroid migration assay (Truong et al., 2012). PC-3 cells treated with either control or EphA2/EphA4 siRNAs were injected into collagen gels. After 6 days, control siRNA-treated cells migrated out of the cancer cell mass into the surrounding collagen gel but EphA2/EphA4 knockdown cells displayed significantly less invasion (Fig. 1H,I). This is consistent with our hypothesis that EphA receptor-mediated contact repulsion enhances cancer cell migration away from neighbouring cancer cells into free space, which could enhance their local dissemination from a primary tumour mass. We next investigated the signalling mechanisms downstream of EphA receptors that mediate contact repulsion behaviour.

### RhoA is required for prostate cancer cell–cell repulsion

**Fig. 2.**
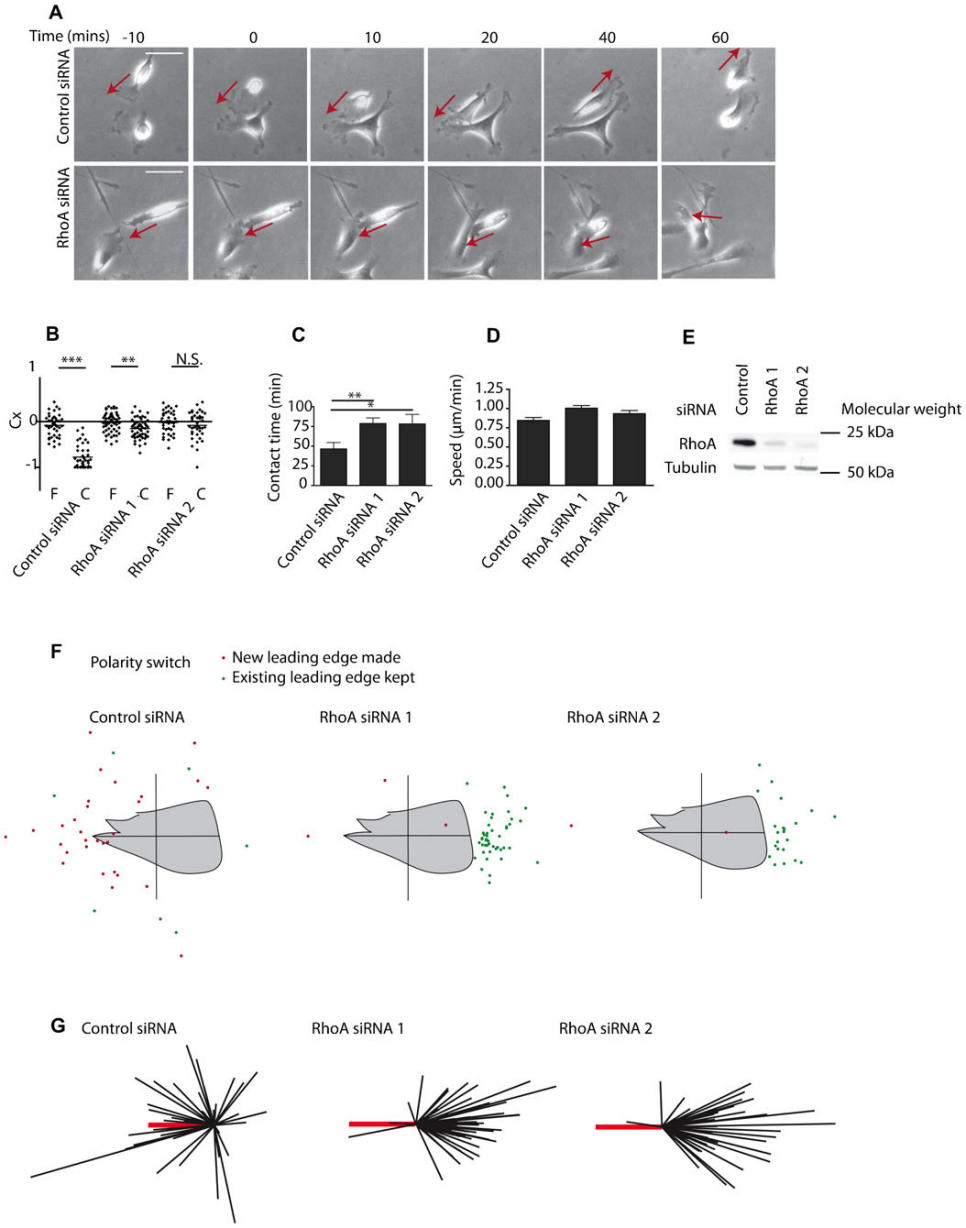
RhoA is required for prostate cancer cell–cell repulsion. (A) Representative images at the indicated timepoints from time-lapse movies of PC-3 cells treated with control or RhoA siRNA (supplementary material Movies 3 and 4). Cells were serum starved and treated with HGF 24 h prior to analysis of cell–cell collisions. 0 min is the time at which cells came into contact. (B) Contact acceleration indices (Cx) of free-moving (F) versus colliding (C) PC-3 cells transfected with control or RhoA siRNA oligonucleotides. (C) Quantification of contact time of PC-3 cells treated with the indicated siRNA oligonucleotides. (D) Quantification of migratory speed. (E) Immunoblotting to show knockdown efficiency with two RhoA siRNA oligonucleotides. (F) Repolarisation diagrams showing the position of newly formed leading edges (red spots) or maintenance of existing leading edges (green spots) after cell–cell collision. (G) Scaled cell-displacement vector diagrams of colliding cells. Scaled displacement of all cells before contact (thick red line) and individual cells after contact (black lines). Triple asterisks indicate P<0.001, two asterisks P<0.01, one asterisk P<0.05, N.S.; not significant, determined by a Mann–Whitney test. Data are from at least three independent experiments. Scale bars: 50 µm.

We have previously shown that ephrin-A5/Fc stimulation activates RhoA downstream of EphA receptors (Astin et al., 2010). EphA receptor activation has been shown to lead to cell retraction via RhoA in several cell types (Astin et al., 2010; Lawrenson et al., 2002; Ogita et al., 2003; Shamah et al., 2001; Wahl et al., 2000) and RhoA has been implicated in CIL (Anear and Parish, 2012; Carmona-Fontaine et al., 2008; Kadir et al., 2011; Theveneau et al., 2010). However, the functional requirement for RhoA in prostate cancer contact repulsion has not been shown. To test this we have analysed CIL using 2D *in vitro* collision assays as previously described (Astin et al., 2010; Paddock and Dunn, 1986). Stills from timelapse movies show that whilst contacting control cells moved apart from one another, RhoA siRNA-treated PC-3 cells did not change direction after contact but rather kept moving in the same direction regardless of cell–cell contact (Fig. 2A; supplementary material Movies 3 and 4). This correlated with reduced repulsion of contacting RhoA siRNA-treated cells when compared to control siRNA-treated cells (Fig. 2B). RhoA siRNA-treated cells also spent increased time in cell–cell contact (Fig. 2C). To investigate this further, repolarisation after cell–cell contact was analysed by tracking the formation of the new leading edge following contact (Fig. 2F). These data show that the majority of control siRNA-treated cells formed a new leading edge away from the point of contact (red dots, Fig. 2F), whereas RhoA siRNA-treated cells tended to keep their existing leading edge and to maintain their original polarity (green dots, Fig. 2F). The data are also shown as acceleration vector diagrams (Fig. 2G), highlighting that most control cells were repelled from one another after collision, whereas RhoA knockdown cells did not switch their direction of migration after contact. RhoA protein levels were reduced with both siRNA oligonucleotides (Fig. 2E). These data demonstrate that RhoA is required for prostate cancer cells to respond to contacts with their immediate cancer cell neighbours and move away from them. We next investigated how RhoA is activated downstream of EphA receptors.

### Vav2 activates RhoA to mediate prostate cancer cell–cell repulsion

**Fig. 3.**
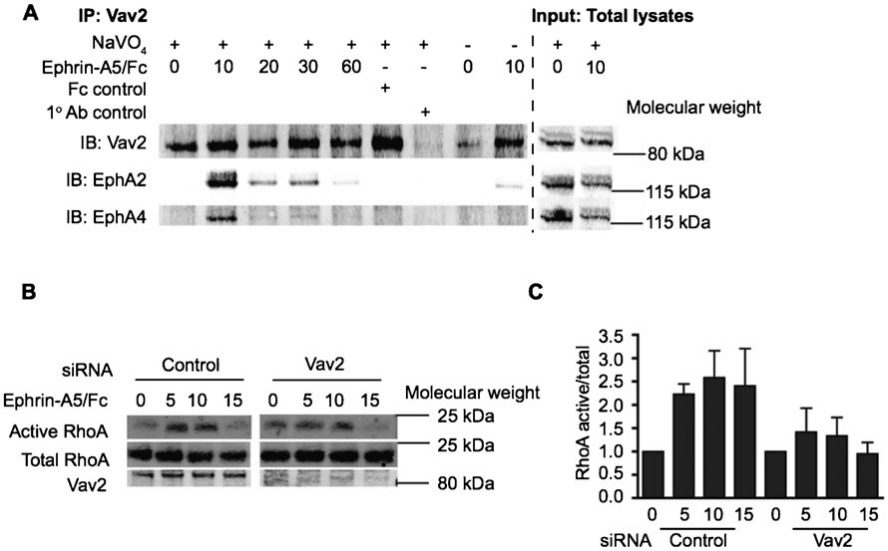
Vav2 is required for ephrin-A5/Fc-induced RhoA activation. (A) PC-3 cells were treated with clustered ephrin-A5/Fc or Fc control and lysed after the indicated time-points with or without NaVO_4_ to inhibit tyrosine phosphatases. Vav2 was immunoprecipitated (IP) from lysates and immunoblotted (IB) for Vav2, EphA2 and EphA4 on immunoprecipitated lysate and on total input lysate. (B) PC-3 cells treated with ephrin-A5/Fc were lysed at the indicated time-points, followed by pulldown of RhoA GTP using Rhotekin Rho-binding domain beads. Proteins were resolved by SDS-PAGE and detected by immunoblotting. (C) Quantification of active RhoA relative to total RhoA. The intensity of the RhoA pulldown band was quantified using the LiCor Odyssey software relative to the RhoA intensity of total cell lysates for each timepoint to normalise active/total RhoA. Each timepoint is displayed relative to the untreated control (0 min) for control and Vav2 siRNA. Means and standard error of the means are shown. Cells were serum starved and treated with HGF 24 h prior to ephrin-A5/Fc stimulation.

The mechanism of RhoA activation downstream of EphA receptors in prostate cancer cells is unknown but a number of GEFs such as Ephexin or Vav have been shown to be required for EphA-induced growth cone collapse, an event that closely resembles cell retraction during CIL (Cowan et al., 2005; Shamah et al., 2001). Vav2 has been shown to activate RhoA downstream of growth factor receptors (Abe et al., 2000; Liu and Burridge, 2000) and is implicated in EphA-mediated axon retraction (Cowan et al., 2005). Here we show that endogenous Vav2 interacts with EphA receptors in prostate cancer cells (Fig. 3A). Following ephrin-A5/Fc treatment, EphA2 and EphA4 immunoprecipitated with Vav2 and this interaction was greatly reduced in the absence of phosphatase inhibitors, suggesting that Vav2 is recruited to and interacts with activated EphA receptors (Fig. 3A). Pulldown assays with the Rho-binding domain of Rhotekin confirmed our previous finding that ephrin-A5/Fc stimulation enhanced RhoA activity (Astin et al., 2010). Vav2 siRNA-treated cells exhibit reduced RhoA activity following ephrin-A5/Fc stimulation compared with control cells (Fig. 3B,C), showing that Vav2 was required for ephrin-A5/Fc induced RhoA activation.

**Fig. 4.**
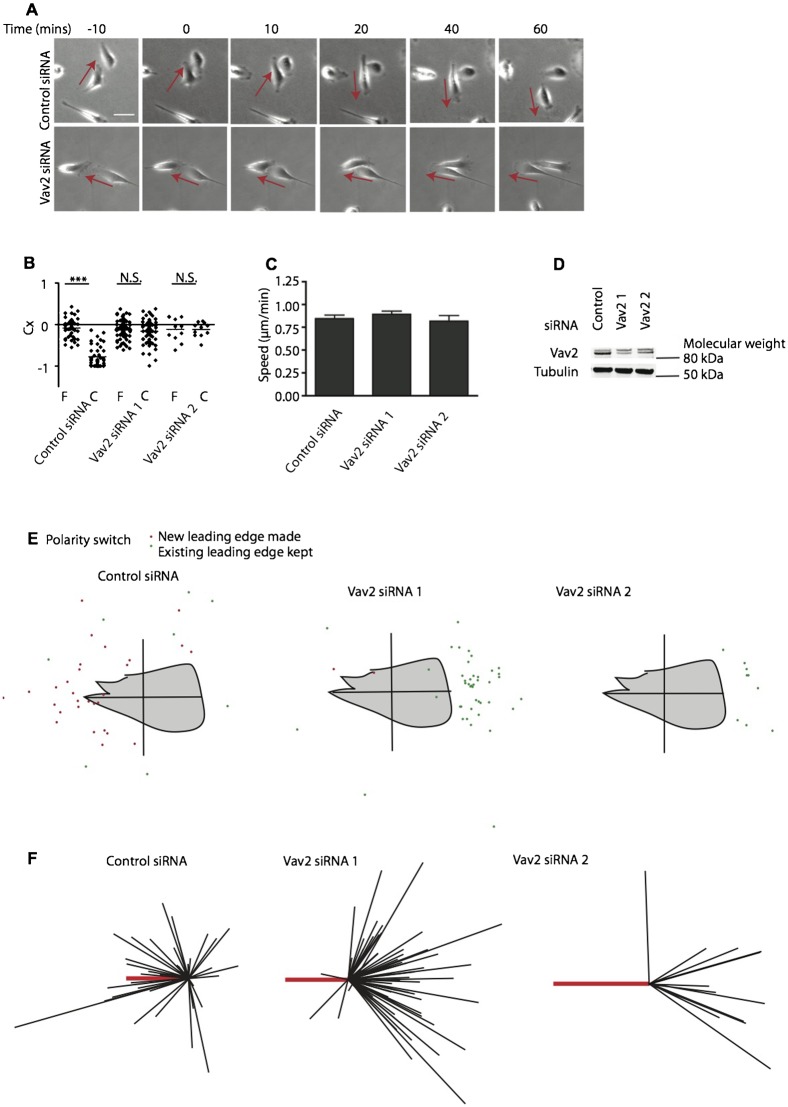
Vav2 mediates prostate cancer cell–cell repulsion. (A) Representative images from time-lapse movies of PC-3 cells treated with control or Vav2 siRNA (supplementary material Movies 5 and 6). Cells were serum starved and treated with HGF 24 h prior to analysis of cell–cell collisions. (B) Contact acceleration indices (Cx) of free moving (F) and colliding (C) cells treated with control siRNA or Vav2 siRNA. (C) Quantification of migratory speed. (D) Immunoblotting shows knockdown efficiency with two Vav2 siRNA oligonucleotides. (E) Repolarisation diagrams showing the position of newly formed leading edges (red spots) or maintenance of existing leading edges (green spots) after cell–cell collision. (F) Scaled cell-displacement vector diagrams of colliding cells. Scaled displacement of all cells before contact (thick red line) and individual cells after contact (black lines). One asterisk indicates P<0.05, triple asterisks indicate P<0.001, N.S.; not significant, determined by a Mann–Whitney test for Cx values and a Student's t-test for contact time. Data are from at least three independent experiments. Scale bar: 50 µm.

We next tested whether Vav2 is required for prostate cancer cell–cell repulsion. Stills from timelapse images show that like RhoA knockdown cells, Vav2 siRNA-treated cells also failed to display cell–cell repulsion (Fig. 4A; supplementary material Movies 5 and 6). This correlated with no significant difference in Cx values between free moving and contacting cells (Fig. 4B) indicating lack of repulsion. There were no significant differences in speed of migration in cells treated with control or Vav2 siRNA (Fig. 4C). Immunoblotting shows knockdown of Vav2 in Vav2 siRNA-treated cells (Fig. 4D). Repolarisation plots show that the majority of control cells formed a new leading edge away from the point of contact, whereas Vav2 knockdown cells kept their original leading edge and maintained migration polarity after collision (Fig. 4E). The data are also shown as vector diagrams (Fig. 4F), highlighting that most control cells are repelled by cell–cell contact, whereas, like RhoA knockdown cells, Vav2 knockdown PC-3 cells continued migrating in the same direction regardless of whether they made contact with another PC-3 cell or not.

### EphA, Vav2 and RhoA signalling affects stability of the microtubule cytoskeleton

**Fig. 5.**
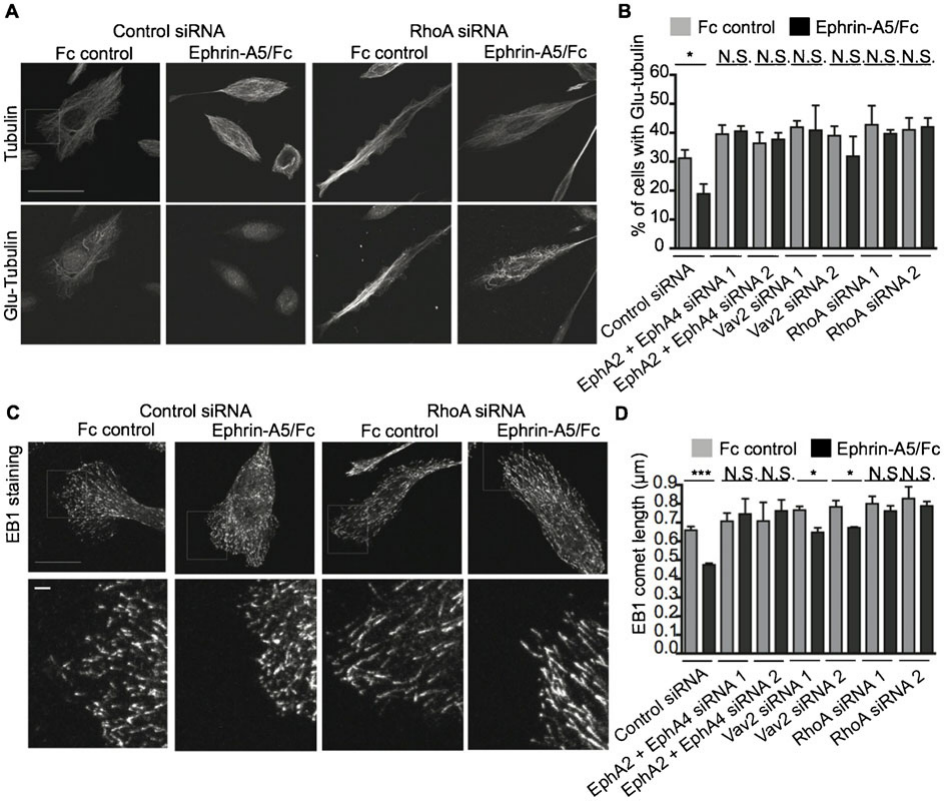
EphA, Vav2 and RhoA signalling affects microtubule stability. (A) Representative confocal images of total tubulin or stable tubulin (Glu-tubulin) in PC-3 cells treated with control or RhoA siRNA and ephrin-A5/Fc or Fc control. (B) Quantification of the % of cells with stable microtubules with the indicated treatments. (C) Representative images of staining for the microtubule tip marker EB1 in control or RhoA siRNA cells treated with ephrin-A5/Fc or Fc control. (D) Quantification of the EB1 comet length with the indicated treatments. Data are from 3 independent experiments. One asterisk indicates P<0.05, two asterisks indicate P<0.01, three asterisks indicate P<0.001, N.S.; not significant. Cells were serum starved and treated with HGF 24 h prior to ephrin-A5/Fc stimulation. Scale bars: 50 µm (A), 10 µm (C), 2 µm (C, inset).

CIL is a multi-step process involving inhibition of migration in the direction that led to contact, followed by retraction away from the contact point, repolarisation and finally, re-initiation of migration in a new direction. It is unknown which of these processes are affected by EphA–Vav2–RhoA signalling during contact repulsion. Due to the known roles of Eph receptors and RhoA signalling to the actin cytoskeleton in mediating cell retraction, it might be presumed that actomyosin contraction is required for CIL to occur. However, previous work in fibroblasts, and here in prostate cancer cells (supplementary material Fig. S1; Movies 11 and 12), showed that inhibition of actomyosin contractility does not inhibit CIL (Kadir et al., 2011). Rather, increased microtubule dynamics are required for the switch in polarity during CIL between contacting fibroblasts and neural crest cells (Kadir et al., 2011; Moore et al., 2013). Stabilisation of microtubules using low doses of taxol leads to a failure of prostate cancer CIL (Batson et al., 2013). We hypothesise that EphA–Vav2–RhoA signalling might increase microtubule destabilisation to enable direction-switching after cell–cell contact. Immunocytochemistry staining of the stable form of tubulin (detyrosinated tubulin or Glu-tubulin) shows that ephrin-A5/Fc treatment decreased the percentage of cells with stable microtubules by comparison to that in control cells (Fig. 5A,B). Furthermore, EphA2 and EphA4, Vav2 and RhoA were all required for this ephrin-A5/Fc induced destabilisation of microtubules since the reduction in percentage of cells with stable microtubules following ephrin-A5/Fc stimulation was not seen in cells treated with EphA2/EphA4, Vav2 or RhoA siRNA. Similarly, staining for the +TIP marker EB1, which binds specifically to polymerising microtubules, shows that ephrin-A5/Fc treatment decreased the length of EB1 comets, indicating reduced microtubule polymerisation following ephrin-A5/Fc stimulation (Bieling et al., 2007). Ephrin-A5/Fc induced reduction in comet length was decreased in PC-3 cells treated with EphA2/EphA4, Vav2 or RhoA siRNAs compared to control siRNA-treated cells (Fig. 5C,D). This is consistent with the hypothesis that EphA signalling via Vav2 and RhoA leads to destabilisation of microtubules and allows cells to repel after contact with one another.

### Partial destabilisation of microtubules restores cell–cell repulsion in EphA2/EphA4, Vav2 and RhoA siRNA-treated PC-3 cells

**Fig. 6.**
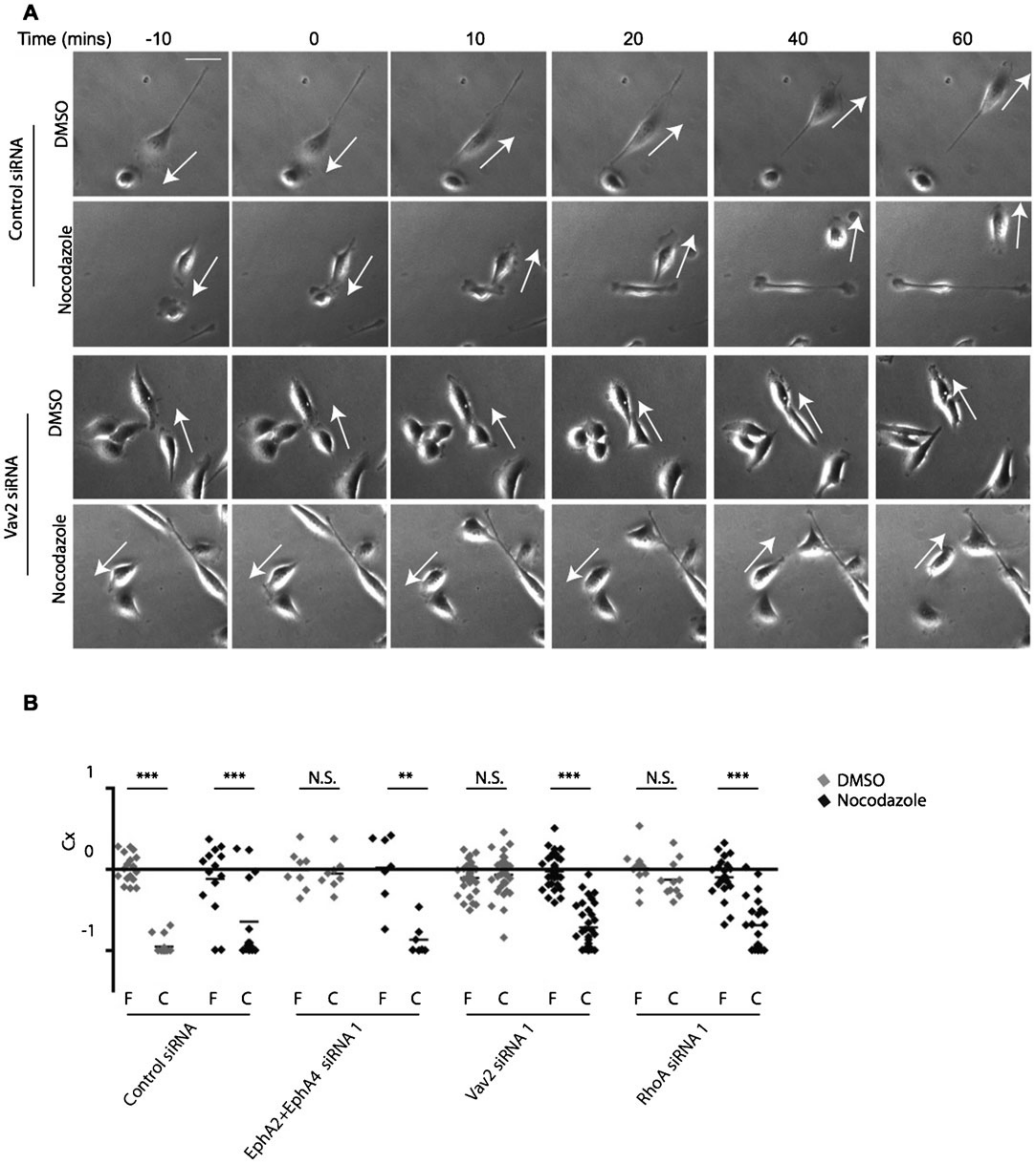
Partial destabilisation of microtubules rescues cell–cell repulsion in EphA2/EphA4, Vav2 or RhoA siRNA-treated PC-3 cells. (A) Representative stills from time-lapse movies of PC-3 cells treated with control or Vav2 siRNA and DMSO or Nocodazole (10 nM) (supplementary material Movies 7–10). Cells were serum starved and treated with HGF 24 h prior to analysis of cell–cell collisions. (B) Contact acceleration indices (Cx) of free-moving (F) versus colliding (C) cells with the indicated siRNA treatments and DMSO or Nocodazole treatment. Two asterisks indicate P<0.01, triple asterisks indicate P<0.001, N.S.; not significant, determined by a Mann–Whitney test. Data are from at least three independent experiments. Arrows indicate the direction of migration. Scale bar: 50 µm.

Since microtubule dynamics are required for prostate cancer cell repulsion and EphA, Vav2 and RhoA knockdown prevented ephrin-A5/Fc induced loss of stable and polymerising microtubules, we hypothesised that the failure of CIL we observed in cells treated with EphA, Vav2 or RhoA siRNA could be due to microtubule hyper-stabilisation. We found that treatment with low doses of the microtubule-destabilising drug nocodazole (which partially destabilises microtubules without inhibiting cell migration (Kadir et al., 2011)) could restore cell repulsion in cells treated with EphA2/EphA4, Vav2 or RhoA siRNA (Fig. 6; supplementary material Movies 7–10). Repulsion was not significantly affected by nocodazole treatment in control cells (Fig. 6). These data indicate that EphA/Vav2/RhoA signalling could enhance microtubule dynamics to enable local cell–cell repulsion.

## DISCUSSION

In this study we have investigated the role of EphA receptor-mediated cell–cell repulsion in cancer cell dissemination and the underlying signalling mechanisms. We show that EphA receptors enhance prostate cancer cell dispersal in 2D and in 3D. Additionally, we find that ephrin-A stimulation of EphA receptors activates RhoA through the GEF Vav2 to destabilise the microtubule cytoskeleton and facilitate cell–cell repulsion.

The stopping of the continued migration of a cell in the same direction following contact with another cell was first described almost 60 years ago by Abercrombie and Heaysman, who defined it as contact inhibition of locomotion (CIL) (Abercrombie, 1970; Abercrombie and Heaysman, 1954). Importantly, Abercrombie and Heaysman observed that collisions between migrating cells lead to a change in the direction of migration away from the point of contact, meaning that rather than completely inhibiting further migration, contacting cells divert their migratory paths away from each other and into free space. In this way, CIL has recently been shown to contribute to the correct embryonic dispersal of several cell lineages including neural crest cells in *Xenopus* and haemocytes in *Drosophila* (Carmona-Fontaine et al., 2008; Davis et al., 2012; Moore et al., 2013). Consistent with the finding that CIL drives embryonic cell dispersal during development, we show here that EphA receptors mediate CIL via Vav2 and RhoA and that EphA2/EphA4 can regulate cancer cell dissemination from dense cancer cell populations.

EphA2 receptor expression has been linked to aggressive progression of prostate, breast, pancreatic, colon, lung cancer and melanoma (Brantley-Sieders et al., 2008; Duxbury et al., 2004; Fang et al., 2005; Margaryan et al., 2009; Taddei et al., 2009; Walker-Daniels et al., 1999; Wykosky and Debinski, 2008). EphA4 has also been found to be expressed at higher levels in prostate cancer cells compared to normal prostate epithelium and has been suggested to play a tumour promoting role in prostate cancer progression (Ashida et al., 2004). EphA2 overexpression in prostate cancer cells has been shown to increase migration via activation of Src and RhoA (Fang et al., 2008) or via Akt (Miao et al., 2009) in a ligand-independent manner. EphA2 can also increase metastatic growth via effects on cell migration (Taddei et al., 2011). We suggest that an additional mechanism, that could explain the association of EphA receptor expression with poor patient prognosis, could be increased cancer cell dispersal due to EphA-mediated cell–cell repulsion.

Our findings are consistent with a recent study showing that MT1-MMP cleavage of EphA2 can enhance individual cancer cell invasion via RhoA-mediated repulsive signalling (Sugiyama et al., 2013). This study suggested that cleavage and endocytosis of EphA2 by MT1-MMP expressed on single breast cancer cells is required for cell–cell repulsion. Overexpression of mutant EphA2, vulnerable to cleavage, led to cell–cell repulsion only when MT1-MMP was also expressed. It is not known whether MT1-MMP cleavage of EphA2 plays a role in CIL during collisions between cells that are already migrating individually or whether this cleavage increases at sites of cell–cell contact between individually migrating cells. Endocytosis has also been shown to be required to terminate Eph–ephrin interactions and allow separation of contacting cells (Marston et al., 2003). Interestingly, the initial work identifying Vav2 as a GEF that interacts with EphA receptors, showed that ephrin triggered axon retraction requires Vav2 regulated endocytosis of Eph receptors (Cowan et al., 2005). Vav2 has been shown to activate Cdc42, Rac and RhoA downstream of growth factor receptors (Abe et al., 2000; Liu and Burridge, 2000). However, the activity of Vav2 towards different Rho GTPases downstream of EphA receptors is unknown. Previous work in our laboratory showed there was an increase in RhoA activity following ephrin-A5/Fc stimulation (Astin et al., 2010). Here we find that Vav2 is required for RhoA activation downstream of EphA receptors and EphA receptor–RhoA-mediated CIL in prostate cancer cells.

Here, we have analysed contact repulsion of individual migrating prostate cancer cells just as might be found in patient high-grade prostate cancer where epithelial organisation is lost (Bostwick et al., 1995b). In less advanced cancer cells and non-cancer cells, it is possible that other molecules may be involved in cell repulsion episodes (Bostwick et al., 1995a; Bracke et al., 1997; Huttenlocher et al., 1998; Takai et al., 2008; Theveneau et al., 2010). Prostate cancer cells must dissolve their epithelial junctions before they can break away from the primary tumour and undergo local single-cell invasion where EphA-mediated repulsion may then be an important driver of dissemination. In this study, as previously published (Astin et al., 2010), prostate cancer cells were serum starved and treated with HGF 24 h prior to all assays, to promote cell migration. The role of HGF in EphA-mediated CIL is unknown. HGF promotes cell scattering and as such could contribute to EphA-mediated dispersal and previous studies have suggested that HGF could enhance EphA-mediated cell behaviour (Miao et al., 2009; Vaught et al., 2009). As the receptor for HGF, c-MET, has been shown to be upregulated in androgen-insensitive and metastatic prostate cancer cells (Humphrey et al., 1995), it would be interesting in future work to test whether this is linked to increased invasiveness via EphA receptor-mediated CIL.

Several studies have shown that Rho GTPases play key roles during CIL (Anear and Parish, 2012; Astin et al., 2010; Carmona-Fontaine et al., 2008; Kadir et al., 2011; Theveneau et al., 2010). Rho GTPases are key regulators of the actin and microtubule cytoskeleton and cell polarity, and may regulate all of the key repulsion steps during CIL. EphA receptors and RhoA are generally thought to mediate cell retraction via actomyosin contractility and it was believed that this provides the mechanism for cell retraction from cell–cell contacts. However, blocking actomyosin contraction with the myosin-II ATPase inhibitor blebbistatin (Kovács et al., 2004; Straight et al., 2003) had no significant effect on cell–cell repulsion in our study (supplementary material Fig. S1; Movies 11 and 12), and in previous work (Kadir et al., 2011). Instead, recent studies showed that microtubule dynamics increase at points of cell–cell contact and are required for the front–rear switch in polarity during CIL (Kadir et al., 2011; Moore et al., 2013), and that stabilisation of microtubules leads to a failure of cell–cell repulsion during cell confrontation (Batson et al., 2013). Here we show that partial destabilisation of microtubules can rescue the failure of repulsion seen with EphA2/EphA4, Vav2 or RhoA knockdown. EphA2/EphA4, Vav2 and RhoA are required for ephrin-A5/Fc induced reduction in the percentage of cells with stable microtubules and in reduction of EB1 comet length. Therefore, we suggest that EphA2 and EphA4 can signal via Vav2 and RhoA leading to microtubule destabilisation and subsequently cell–cell repulsion, although we cannot rule out additional signalling events regulating cancer cell repulsion downstream of EphA receptors.

Microtubules assemble into a polarised array in migrating cells and stable microtubules extend to the leading edge in the direction of cell migration (Stramer et al., 2010; Watanabe et al., 2005; Wittmann and Waterman-Storer, 2001). Several studies have shown that microtubule stabilisation promotes directionally persistent migration (Gundersen and Bulinski, 1988; Redd et al., 2006; Stramer et al., 2010; Watanabe et al., 2005; Wen et al., 2004). Although RhoA has been shown to stabilise microtubules, several studies have found that stable microtubules are depleted from points of cell–cell contact (Gundersen and Bulinski, 1988; Kadir et al., 2011; Nagasaki et al., 1992), where RhoA has also been shown to be active (Carmona-Fontaine et al., 2008). It is not currently clear how RhoA activity might lead to microtubule destabilisation. In neuroblastoma cells, RhoA activation results in increased phosphorylation of the microtubule associated protein, tau, which could promote its dissociation from microtubules and result in their destabilisation (Sayas et al., 1999). Additionally microtubule destabilisation has been shown to regulate RhoA activity (Chang et al., 2008; Ren et al., 1999), suggesting that feedback loops could operate. In this study, we have analysed the percentage of cells with Glu-tubulin as a global indication of microtubule stability, and EB1 comet length as an indication of the level of growing rather than shrinking microtubules. Although our data do not examine which aspect of microtubule stability might be affected by EphA–Vav2–RhoA signalling, our finding that ephrin-A5/Fc stimulation reduces the percentage of cells with Glu-tubulin is consistent with previous work showing that Glu-tubulin is lost from points of cell–cell contact (Nagasaki et al., 1992). Our data showing that EB1 comet length is decreased following ephrin-A5/Fc stimulation are also consistent with data that show that microtubule dynamics increase at points of cell–cell contact (Kadir et al., 2011; Moore et al., 2013).

We propose that EphA–Vav2–RhoA-mediated repulsion between contacting cancer cells at the tumour margin could enhance their local invasion away from the primary tumour.

## MATERIALS AND METHODS

### Cell culture and reagents

PC-3 cells were maintained as previously described (Astin et al., 2010). Cells were serum starved for 24 h and then treated with HGF (10 ng ml^−1^) overnight prior to all assays to stimulate cell migration.

Ephrin-A5/Fc was obtained from R&D systems. HGF was obtained from Peprotech. Blebbistatin was from Tocris and Nocodazole was from Sigma (supplementary material Table S3). The following antibodies were used; anti-Glu tubulin (rabbit polyclonal, Chemicon), anti-Tyr tubulin (rat polyclonal, AbD Serotec), anti-EB1 (mouse monoclonal, BD Transduction laboratories), anti-RhoA (monoclonal mouse, Cytoskeleton), anti-Vav2 (rabbit polyclonal, Santa Cruz), anti-EphA2 (mouse monoclonal, Upstate), anti-cortactin was used as a primary antibody control for Vav2 immunoprecipitation (mouse monoclonal, Upstate), anti-phosphotyrosine 4G10 (mouse monoclonal, Millipore). EphA4 antibody was a kind gift from David Wilkinson (NIMR, UK). Antibody dilutions are given in supplementary material Table S2.

### Time-lapse imaging and confocal microscopy

Time-lapse microscopy imaging was performed on an inverted Zeiss microscope with an Orca-ER camera (Hamamatsu) and Improvision software. To record large numbers of cell–cell collisions, phase-contrast time-lapse microscopy was performed on an inverted Leica DMIRE microscope equipped with a Maerzhaeuser scanning stage for multi-position imaging, at 37°C, and cells were maintained in CO_2_-independent medium (Invitrogen). An image was taken every 5 min using a 10× objective. Image analysis was performed using Volocity software (Improvision) and Image J.

Confocal microscopy of fixed cells was performed on a Leica SP5 confocal microscope. For microtubule staining, cells were fixed in ice cold methanol for 4 min, rehydrated in PBS and blocked with 1% BSA (Sigma) in PBS for 1 h at room temperature. Cells were incubated with primary antibodies (in 1% BSA/PBS) for 1 h and bound antibodies were detected using appropriate fluorescence-conjugated secondary antibodies for 45 min at room temperature.

### Contact inhibition of locomotion

For analysis of homotypic collisions, 3,000–4,000 cells were grown on 13 mm glass coverslips coated with matrigel (diluted 1 in 3 with culture medium; Sigma) for 10 h. Quantification of CIL was carried out as described previously (Astin et al., 2010; Paddock and Dunn, 1986). Briefly, the displacement of a migrating cell for 50 min before collision (vector A) and for 50 min following collision (vector B) was measured. The component Cx of vector B–A represents the difference between how far the cell has progressed in the direction of A′ and how far it would have gone had there been no collision. Cx values were also calculated for the same population of cells that were free-moving and not colliding, by tracking cell movements over the same time periods. CIL was considered to have occurred when the mean Cx value of free-moving cells was significantly different to that of colliding cells as determined using a Mann–Whitney U-test. Cx measurements were scaled to normalise for differences in speed between cell populations. Cell speed, contact time and repolarisation were analysed as previously described (Kadir et al., 2011).

For 2D dispersal assays PC-3 cells were seeded into silicon inserts (Ibidi), serum starved and treated with HGF after 24 h and inserts were removed and cells imaged after 48 h. Cells were tracked for 24 h from the edge of the cancer cell population using Volocity software. The total distance of migration and the direct distance between the first and last point were measured.

### Cell stimulation, immunoprecipitation and Western blotting

Serum-starved PC-3 cells, cultured on 6 cm dishes or matrigel-coated coverslips, were treated with ephrin–Fc chimeras (1 µg/ml) pre-clustered for 20 min at 37°C with goat anti-human-Fc antibody (10 µg/ml, Stratech). For Western blotting, cells were serum starved at 50% confluence, and treated with 10 ng/ml HGF. Cells were washed once in cold PBS before lysing in RIPA buffer for 10 min on ice. Lysates were vortexed for 10 min, centrifuged and the supernatant retained.

The levels of Rho GTPase–GTP were measured using a RhoA GTP pulldown kit (Cytoskeleton), according to the manufacturer's instructions. Bands were quantified and normalised intensities expressed relative to 0 min control (assigned as 1).

For immunoprecipitation, ephrin-A5/Fc or Fc stimulated cells were lysed in immunoprecipitation lysis buffer (125 mM sodium chloride, 20 mM Tris pH 7.4, 1% Igepal CA-630 (Sigma), 10% glycerol, 1 mM phenylmethanesulphonylfluoride (PMSF), 50 mM sodium orthovanadate, 5 mM sodium fluoride, 1× protease inhibitor cocktail (Roche)). 1 µg primary antibody was added to an equal amount of protein for each sample lysate and rotated at 4°C overnight. Protein A Sepharose beads (Sigma) for rabbit primary antibodies were washed twice and then 8 µl beads were added to each sample. An isotype-matched primary antibody was used as a negative control. The sample/bead mixture was rotated at 4°C for 1 h, washed 3 times, and resuspended in 2× protein sample buffer. Proteins were resolved by SDS-PAGE and detected by immunoblotting.

### siRNA

Between 10–25 nM siRNA oligonucleotides (Dharmacon) were transfected into PC-3 cells using RNAi max (LifeTech). Transfected cells were imaged between 72 and 96 h post-transfection. siRNA oligonucleotide sequences are detailed in supplementary material Table S1. Two different siRNAs were used for each knockdown. For all siRNA experiments, a non-targeting siRNA oligonucleotide was used as negative control.

### Spheroid invasion assay

Collagen I was prepared from rat tails as previously described (Timpson et al., 2011). 1 mm × 0.58 mm glass capillaries were pulled and bevelled to a tip diameter of 60 µm. Gels were mixed on ice, containing 3 parts collagen + 1 part NaHCO_3_ + 1 part 10× RPMI + 1 part 1 M Hepes + 8 parts deionised H_2_O. Gels were aliquoted into 4 well dishes (Nunc) and allowed to set at 37°C for 1 h. During this time, cells were trypsinised, washed in PBS and resuspended in 2% polyvinylpyrrolidone (PVP). Cells were incubated in PVP for 20 mins and then injected into set collagen gels using a Picospritzer III (Parker Hannifin) injector and a Leica upright light microscope with a 5 msec injection time at 20 psi (138 kPa). Gels were covered with 0.5% RPMI, which was changed daily. Images were taken at the indicated timepoints and at the endpoint gels were fixed in 10% paraformaldehyde and stained with phalloidin. Cancer cell invasion was measured by quantifying the invasion index from the edge of the cancer cell spheroid as previously described (Nyström et al., 2005).

## Supplementary Material

Supplementary Material
