# Supplementary material for: EphA receptors regulate prostate cancer cell dissemination through Vav2–RhoA mediated cell–cell repulsion
Source: Biol Open. 2014 May 2;3(6):453–62. doi: 10.1242/bio.20146601 (PMC4058079; doi:10.1242/bio.20146601)
Supplement: Supplementary Material [file supp_3_6_453__index.html]

EphA receptors regulate prostate cancer cell dissemination through Vav2–RhoA mediated cell–cell repulsion — Supplementary Material 

# EphA receptors regulate prostate cancer cell dissemination through Vav2–RhoA mediated cell–cell repulsion

## bio.20146601 Supplementary Material

**Files in this Data Supplement:**

- Supplementary Material - Jennifer Batson et al. doi: 10.1242/bio.20146601
- Movie 1 - **Movie 1. Phase time-lapse images overlaid with tracking plots to show a migrating control siRNA-treated PC-3 cell colliding with another cell at the edge of the cancer cell population.** Frames taken every 5 mins and displayed at 10 fps.
- Movie 2 - **Movie 2. Phase time-lapse images overlaid with tracking plots to show a migrating EphA2 ± EphA4 siRNA-treated PC-3 cell colliding with another cell at the edge of the cancer cell population.** Frames taken every 5 mins and displayed at 10 fps.
- Movie 3 - **Movie 3. Representative phase time-lapse images of control siRNA-treated PC-3 cells during cell–cell collisions.** Frames taken every 5 mins and displayed at 4 fps.
- Movie 4 - **Movie 4. Representative phase time-lapse images of RhoA siRNA-treated PC-3 cells during cell–cell collisions.** Frames taken every 5 mins and displayed at 4 fps.
- Movie 5 - **Movie 5. Representative phase time-lapse images of control siRNA-treated PC-3 cells during cell–cell collisions.** Frames taken every 5 mins and displayed at 4 fps.
- Movie 6 - **Movie 6. Representative phase time-lapse images of Vav2 siRNA-treated PC-3 cells during cell–cell collisions.** Frames taken every 5 mins and displayed at 4 fps.
- Movie 7 - **Movie 7. Representative phase time-lapse images of control siRNA-treated PC-3 cells treated with DMSO during cell–cell collisions.** Frames taken every 5 mins and displayed at 4 fps.
- Movie 8 - **Movie 8. Representative phase time-lapse images of control siRNA-treated PC-3 cells treated with Nocodazole during cell–cell collisions.** Frames taken every 5 mins and displayed at 4 fps.
- Movie 9 - **Movie 9. Representative phase time-lapse images of Vav2 siRNA-treated PC-3 cells treated with DMSO during cell–cell collisions.** Frames taken every 5 mins and displayed at 4 fps.
- Movie 10 - **Movie 10. Representative phase time-lapse images of Vav2 siRNA-treated PC-3 cells treated with Nocodazole during cell–cell collisions.** Frames taken every 5 mins and displayed at 4 fps.
- Movie 11 - **Movie 11. Representative phase time-lapse images of DMSO-treated PC-3 cells during cell–cell collisions.** Frames taken every 5 mins and displayed at 4 fps.
- Movie 12 - **Movie 12. Representative phase time-lapse images of blebbistatin-treated PC-3 cells during cell–cell collisions.** Frames taken every 5 mins and displayed at 4 fps.
