# Supplementary material for: EphA receptors regulate prostate cancer cell dissemination through Vav2–RhoA mediated cell–cell repulsion
Source: Biol Open. 2014 May 2;3(6):453–62. doi: 10.1242/bio.20146601 (PMC4058079; doi:10.1242/bio.20146601)
Supplement: Supplementary Material [file supp_bio.20146601_bio.20146601-s1.pdf]

Biology Open (2014) 000, 1–10 doi:10.1242/bio.20146601

Supplementary Material
Jennifer Batson et al. doi: 10.1242/bio.20146601

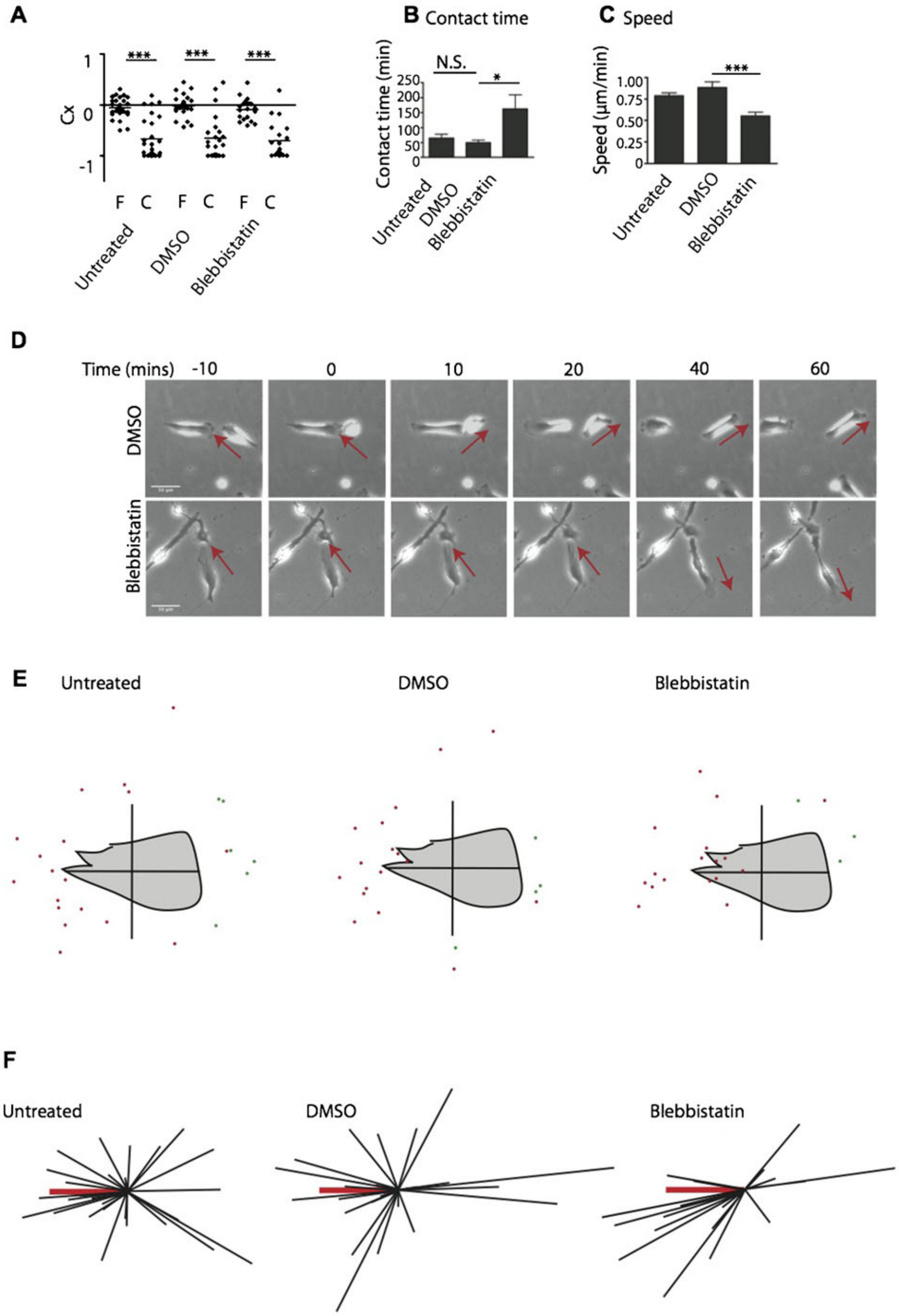

Fig. S1. Actomyosin contractility is not required for cell–cell repulsion. (A) Contact acceleration indices, (B) contact time and (C) speed of PC-3 cells treated with DMSO, or Blebbistatin. (D) Stills from timelapse images. (E) Repolarisation diagrams showing the position of newly formed leading edges (red spots) or maintenance of existing leading edges (green spots) after cell–cell collision. (F) Vector diagrams. Data are from at least three independent experiments. Scale bars: 50 µm.

Biology Open (2014) 000, 1–10 doi:10.1242/bio.20146601

Table S1. siRNA oligonucleotides

siRNA	Final concentration	Catalogue number
EphA2 1	25 nM	J-003116-09
EphA2 2	25 nM	J-003116-10
EphA4 1	25 nM	J-003118-09
EphA4 2	25 nM	J-003118-10
RhoA 1	10 nM	J-003860-11-0005
RhoA 2	10 nM	J-003860-10-0005
Vav2 1	25 nM	J-005199-05-0005
Vav2 2	25 nM	J-005199-07-0005
NT siRNA	25 nM	D-001810-01

All siRNA oligonucleotides were obtained from Dharmacon, Thermo Scientific. Lipofectamine RNAi max transfection reagent (Invitrogen) was used for siRNA delivery.

Table S2. Antibody dilutions

Primary antibody	Species	Dilution	Supplier	Stored at	Use
Polyclonal anti-Glu tubulin	Rabbit	1:250	Chemicon	−20°C	Immunocytochemistry
Monoclonal anti-Tyr tubulin YL1/2	Rat	1:1,000	AbD Serotec	−20°C	Immunocytochemistry
EB1	Mouse	1:500	BD transduction	−20°C	Immunocytochemistry
RhoA	Mouse	1:1,000	Cytoskeleton	4°C	Western
Vav2 (H-200)	Rabbit	1:1,000	Santa Cruz	4°C	Western
EphA2	Mouse	1:1,000	Upstate	−20°C	Western
EphA4	Rabbit	1:5,000	Kind gift from David Wilkinson	4°C	Western
Cortactin	Mouse	1:1,000	Upstate	−20°C	Western
Phospho-tyrosine 4G10	Mouse	1:1,000	Millipore	4°C	Western

Table S3. Inhibitors

Inhibitor	Target	Supplier	Concentration used	Stock concentration
Blebbistatin	Myosin II ATPase	Tocris	50 µM	100 mM in DMSO
Nocodazole	GDP-tubulin	Sigma	10 nM	10 mM in DMSO
Taxol	β subunit of α/β-tubulin dimer	Sigma	5 nM	10 mM in DMSO

Biology Open

Biology Open (2014) 000, 1–10 doi:10.1242/bio.20146601

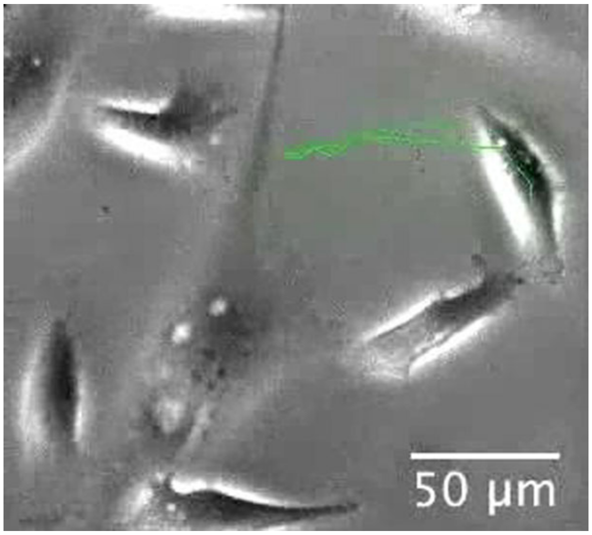

Movie 1. Phase time-lapse images overlaid with tracking plots to show a migrating control siRNA-treated PC-3 cell colliding with another cell at the edge of the cancer cell population. Frames taken every 5 mins and displayed at 10 fps.

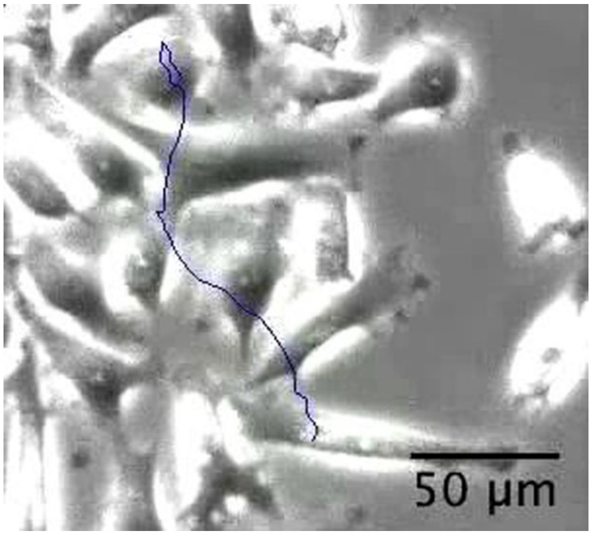

Movie 2. Phase time-lapse images overlaid with tracking plots to show a migrating EphA2 + EphA4 siRNA-treated PC-3 cell colliding with another cell at the edge of the cancer cell population. Frames taken every 5 mins and displayed at 10 fps.

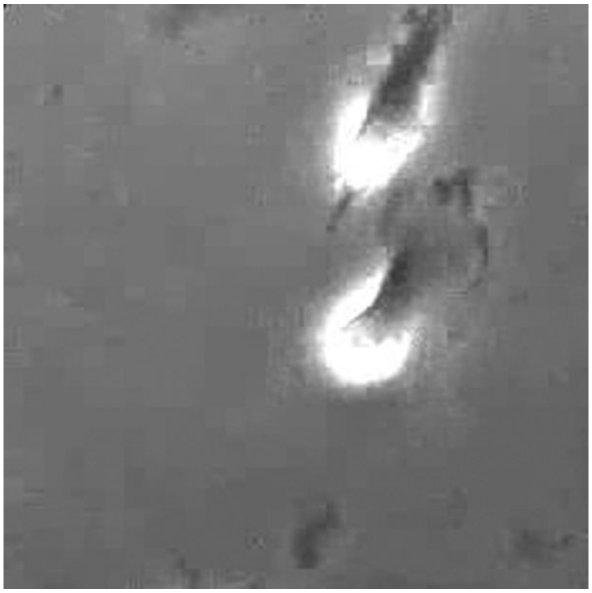

Movie 3. Representative phase time-lapse images of control siRNA-treated PC-3 cells during cell–cell collisions. Frames taken every 5 mins and displayed at 4 fps.

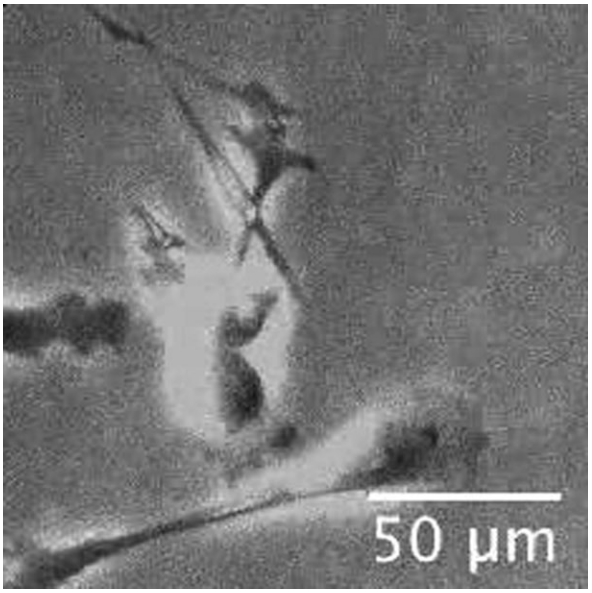

Movie 4. Representative phase time-lapse images of RhoA siRNA-treated PC-3 cells during cell–cell collisions. Frames taken every 5 mins and displayed at 4 fps.

Biology Open

Biology Open (2014) 000, 1–10 doi:10.1242/bio.20146601

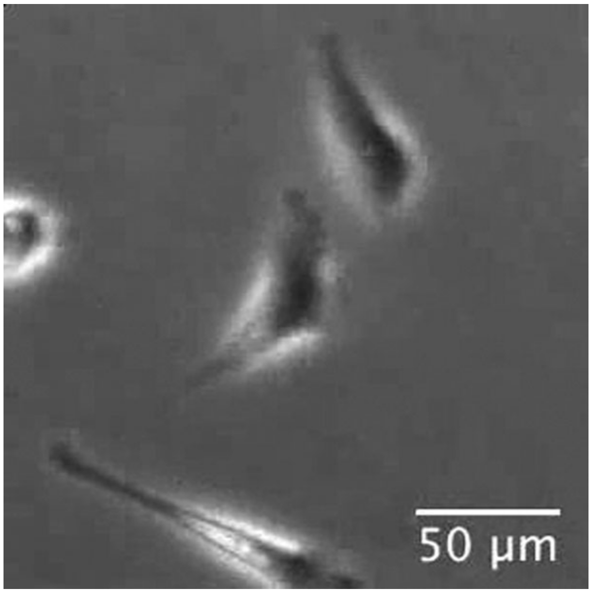

Movie 5. Representative phase time-lapse images of control siRNA-treated PC-3 cells during cell–cell collisions. Frames taken every 5 mins and displayed at 4 fps.

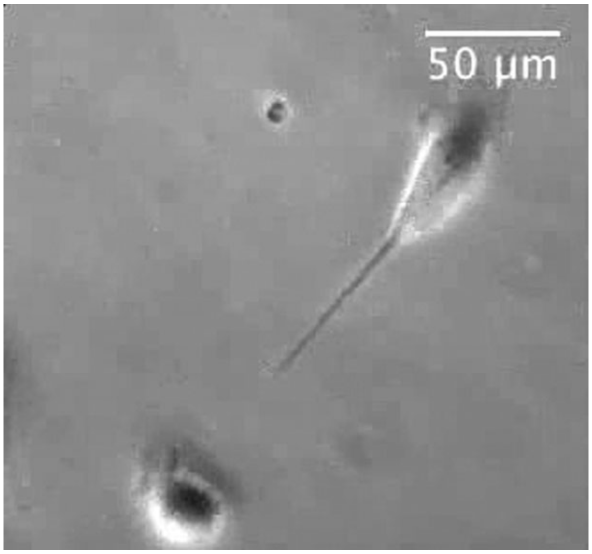

Movie 7. Representative phase time-lapse images of control siRNA-treated PC-3 cells treated with DMSO during cell–cell collisions. Frames taken every 5 mins and displayed at 4 fps.

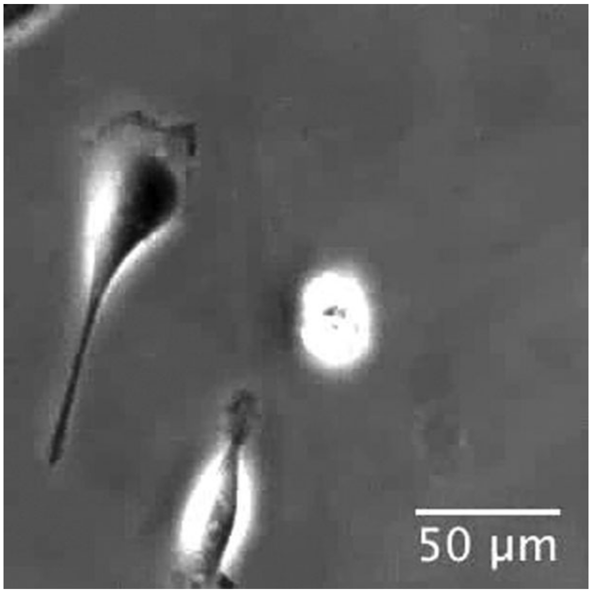

Movie 6. Representative phase time-lapse images of Vav2 siRNA-treated PC-3 cells during cell–cell collisions. Frames taken every 5 mins and displayed at 4 fps.

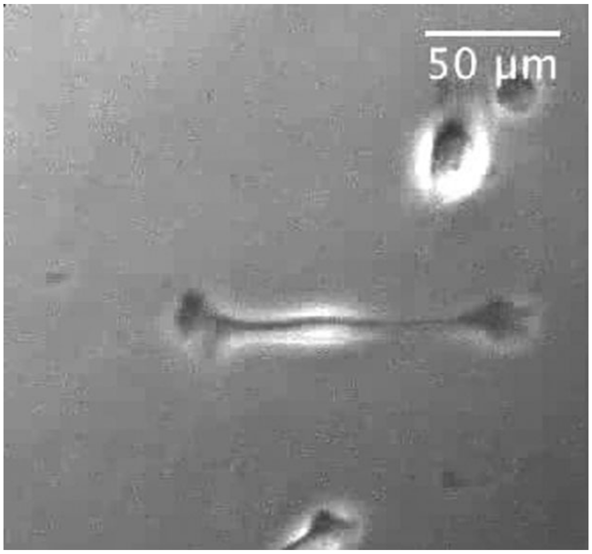

Movie 8. Representative phase time-lapse images of control siRNA-treated PC-3 cells treated with Nocodazole during cell–cell collisions. Frames taken every 5 mins and displayed at 4 fps.

Biology Open

Biology Open (2014) 000, 1–10 doi:10.1242/bio.20146601

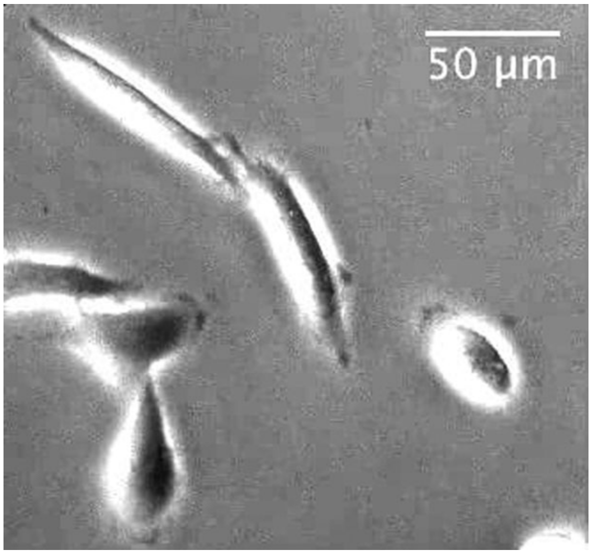

Movie 9. Representative phase time-lapse images of Vav2 siRNA-treated PC-3 cells treated with DMSO during cell–cell collisions. Frames taken every 5 mins and displayed at 4 fps.

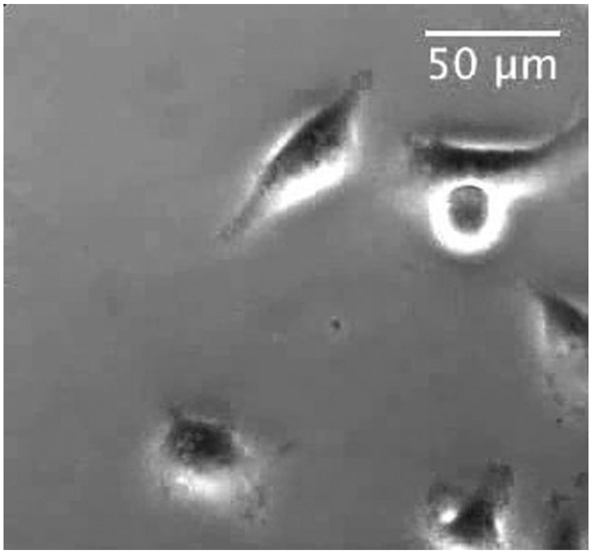

Movie 10. Representative phase time-lapse images of Vav2 siRNA-treated PC-3 cells treated with Nocodazole during cell–cell collisions. Frames taken every 5 mins and displayed at 4 fps.

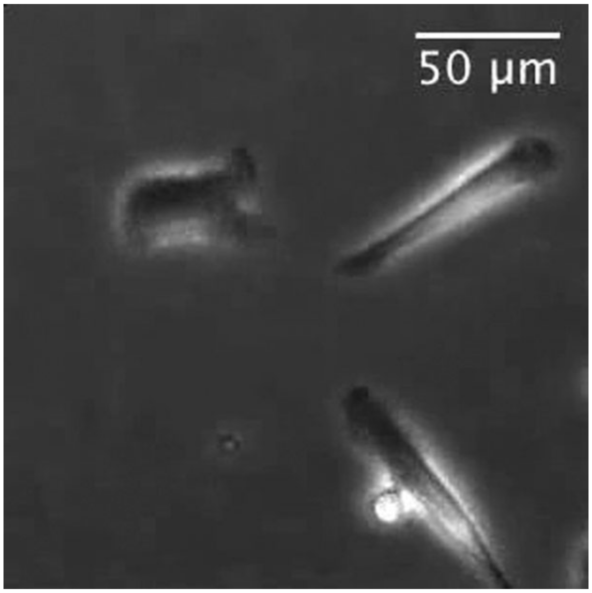

Movie 11. Representative phase time-lapse images of DMSO-treated PC-3 cells during cell–cell collisions. Frames taken every 5 mins and displayed at 4 fps.

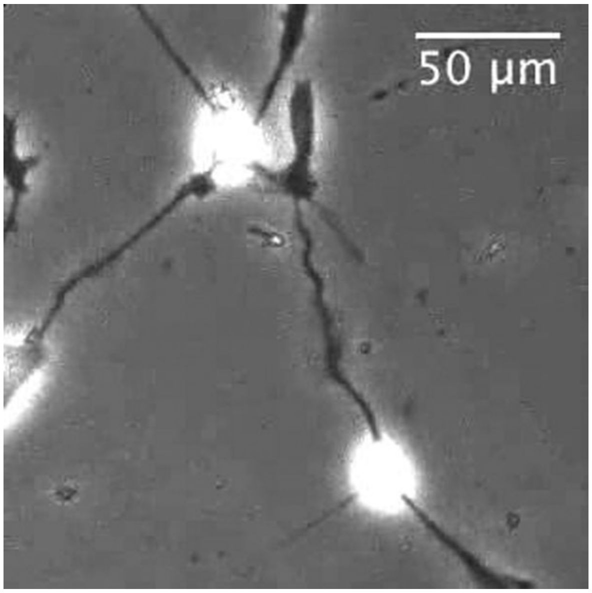

Movie 12. Representative phase time-lapse images of blebbistatin-treated PC-3 cells during cell–cell collisions. Frames taken every 5 mins and displayed at 4 fps.

Biology Open
